# Reviewed article: Feitosa YS, Cabral BVB, Lima GS, Gomes EB, Sousa GJB, Moreira TMM, Pereira MLD. Surgical treatment of diabetic foot: a spatial analysis, Ceará, Brazil, 2016-2021. Epidemiol Serv Saude. 2026:35;e20240755

**DOI:** 10.1590/S2237-96222026v35e20240755.a

**Published:** 2025-12-19

**Authors:** Maria do Carmo Fernandez Lourenço Haddad

**Affiliations:** 1 Universidade Estadual de Londrina, Londrina, PR, Brazil Londrina PR Brazil

## Peer review A

## Questionnaire

Does the manuscript contain new and significant information to justify publication? Yes

Does the Abstract (Summary) clearly and accurately describe the content of the article? Yes

Is the problem significant and concisely stated? Yes

Are the methods described comprehensively? Yes

Are the interpretations and conclusions justified by the results? Yes

Is adequate reference made to other work in the field? Yes

## Manuscript Structure

Length of article is: Adequate

Number of tables is: Adequate

Number of figures is: Adequate

Please state any conflict(s) of interest that you have in relation to the review of this paper. None

Interest: Excellent

Quality: Excellent

Originality: Excellent

Overall: Excellent

Recommendation: Accept

## Comments

O tema de pesquisa é relevante, apresenta rigor metodológico e análises estatísticas robustas.

Referencias bibliográficas atuais.

Sugestões de adequações no texto:

Página 1:

Linha 18 - ...no estado do Ceará, Brasil.

Linha 25 - ...pessoas com diabetes mellitus (DM)...

Linha 26 - ...das internações por DM...

Linha 29 - A capital Fortaleza se destaca...

Linha 58 - ...estado do Ceará, Brasil, registrou...

Página 2:

Linha 8 - ... associados ao diabetes mellitus (DM), como as úlceras podálicas, que quando não diagnosticadas...

Linha 26 a 27 - Inserir entre parênteses as porcentagens encontradas no estado da Bahia e Paraíba e no Cariri.

Linha 46 -...permitindo realizar análise (suprimir a palavra os autores).

Linha 50 - ...saúde na elaboração de estratégias de intervenções preventivas.

* Substituir em todo o texto a palavra “diabetes” pela sigla DM.

Página 3, linha 40 - ...com complicações nos pés sobre...

Página 5:

Linha 21 - ...nº 510/2016...

Linha 24 - nº 466/2012.

linha 31 - ...à Informação (nº 12.527/2011).

Página 36 - Resultados

Uniformizar a localidade de todas as tabelas e figuras, pois as vezes aparece como “...Ceará; outras Fortaleza, Ceará, Brasil..., entre outras formas de apresentação”.

Página 7, linha 57 - Porem, destaca-se que o Nordeste...

